# Genomic insights into a plant growth‐promoting *Pseudomonas koreensis* strain with cyclic lipopeptide‐mediated antifungal activity

**DOI:** 10.1002/mbo3.1092

**Published:** 2020-06-15

**Authors:** Yilin Gu, Yi‐Nan Ma, Jing Wang, Zhenyuan Xia, Hai‐Lei Wei

**Affiliations:** ^1^ Institute of Agricultural Resources and Regional Planning Chinese Academy of Agricultural Sciences Key Laboratory of Microbial Resources Collection and Preservation Ministry of Agriculture and Rural Affairs Beijing China; ^2^ Yunnan Academy of Tobacco Agricultural Science Kunming China

**Keywords:** biological control, Cyclic lipopeptides, non‐ribosomal peptide synthetase, *Pseudomonas koreensis*, secondary metabolite

## Abstract

Strain S150 was isolated from the tobacco rhizosphere as a plant growth‐promoting rhizobacterium. It increased plant fresh weight significantly and lateral root development, and it antagonized plant pathogenic fungi but not phytobacteria. Further tests showed that strain S150 solubilized organic phosphate and produced ammonia, siderophore, protease, amylase, and cellulase, but it did not produce indole‐3‐acetic acid. Using morphology, physiological characteristics, and multi‐locus sequence analysis, strain S150 was identified as *Pseudomonas koreensis*. The complete genome of strain S150 was sequenced, and it showed a single circular chromosome of 6,304,843 bp with a 61.09% G + C content. The bacterial genome contained 5,454 predicted genes that occupied 87.7% of the genome. Venn diagrams of the identified orthologous clusters of *P. koreensis* S150 with the other three sequenced *P. koreensis* strains revealed up to 4,167 homologous gene clusters that were shared among them, and 21 orthologous clusters were only present in the genome of strain S150. Genome mining of the bacterium *P. koreensis* S150 showed that the strain possessed 10 biosynthetic gene clusters for secondary metabolites, which included four clusters of non‐ribosomal peptide synthetases (NRPSs) involved in the biosynthesis of cyclic lipopeptides (CLPs). One of the NRPSs possibly encoded lokisin, a cyclic lipopeptide produced by fluorescent *Pseudomonas*. Genomic mutation of the *lokA* gene, which is one of the three structural NRPS genes for lokisin in strain S150, led to a deficiency in fungal antagonism that could be restored fully by gene complementation. The results suggested that *P. koreensis* S150 is a novel plant growth‐promoting agent with specific cyclic lipopeptides and contains a lokisin‐encoding gene cluster that is dominant against plant fungal pathogens.

## Graphical abstract

We reported a newly isolated *Pseudomonas koreensis* strain S150, which antagonized plant pathogens, solubilized phosphates, produced siderophores and enzymes, and promoted plant growth. Genome mining showed that the strain S150 possesses 10 genomic islands coding for secondary metabolites, one of which is lokisin, an unusual cyclic lipopeptide. We generated a deletion mutant for the smallest non‐ribosomal peptide synthetase gene lokA in strain S150. Antifungal activity was deficient in the lokA mutant and was restored by gene complementation.

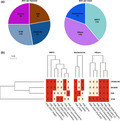

## 1 INTRODUCTION

Plant growth‐promoting rhizobacteria (PGPR), which include soilborne fluorescent *Pseudomonas* strains, confer plant growth‐promoting (PGP) traits on plant health and productivity by antagonizing phytopathogens and by improving the availability and assimilation of nutrients (Fravel, 2005; Haas & Défago, 2005; O'Sullivan & O'Gara, 1992). Fluorescent *Pseudomonas* includes a variety of species that occur in a broad range of ecological niches, such as soils, plant tissues, and the rhizosphere (Haas & Keel, 2003; Yamamoto et al., 2000). In general, PGPR *Pseudomonas* produces antimicrobial compounds, hydrolytic enzymes, and volatiles to inhibit phytopathogens or to induce systemic resistance (ISR) to reduce plant disease (Bonsall, Weller, & Thomashow, 1997; Harrison, Letendre, Kovacevich, Pierson, & Weller, 1993; Keel et al., 1992; Ongena et al., 2004). On the other hand, it promotes plant development through phosphate solubilization and siderophore release or by synthesizing phytohormones such as auxins, cytokinins, gibberellins, and nitric oxide (NO) (Das, Katiyar, & Goel, 2003; Grichko & Glick, 2001; Gyaneshwar, Naresh, Parekh, & Poole, 2002).

Some *Pseudomonas* species, such as *P. fluorescens*, *P. protegens*, *P. kilonensis*, *P. chlororaphis*, and *P. simiae*, have been used widely in agriculture to control plant disease and to improve production (Raaijmakers, Weller, & Thomashow, 1997; Ramamoorthy, Raguchander, & Samiyappan, 2002). Among them, *P. kilonensis* F113, *P. protegens* strains CHA0 and Pf‐5, and some other typical PGPR strains produced polyketides, such as 2,4‐diacetylphloroglucinol (DAPG), phenazines, pyoluteorin, and pyrrolnitrin, that defended against a broad range of plant pathogens (Almario et al., 2017; Iavicoli, Boutet, Buchala, & Métraux, 2003; Keel et al., 1992; Nowak‐Thompson, Gould, & Loper, 1997; Ramette et al., 2011). In addition to polyketides, many PGPR strains synthesized cyclic lipopeptides (CLPs) by non‐ribosomal peptide synthetases (NRPSs) as potent secondary metabolites that can destroy microbial membranes (Geudens & Martins, 2018). Lokisin is a member of the amphisin group of CLPs, which was first isolated from PGPR strain *Pseudomonas* sp. DSS41 in 2002 (Sørensen, Nielsen, Sørensen, & Christophersen, 2002). It is a relatively large lipopeptide with a molecular weight of 1,354.6 g/mol and contains a β‐hydroxydecanoyl moiety and 11 amino acid (AA) residues in the cyclic peptide structure (Sørensen et al., 2002). Isolated lokisin from strain DSS41 and *P. koreensis* 2.74 showed antagonistic activity against some plant fungal pathogens such as *Pythium ultimum* and *Rhizoctonia solani* (Hultberg, Alsberg, Khalil, & Alsanius, 2010; Sørensen et al., 2002). But the biosynthetic gene cluster of lokisin was reported most recently by whole‐genome sequencing of *Pseudomonas* sp. COR10 (Omoboye et al., 2019). Unfortunately, the genome information for strain COR10 is not publicly available to date.

Here, we report the isolation and taxonomic classification of the novel bacterial strain *P. koreensis* S150. The ability of this strain to antagonize plant pathogens and to promote plant growth was determined. The complete genome of strain S150 was sequenced, and genome mining and genetic evidence revealed that a lokisin‐encoding gene cluster was associated with anti‐fungal capacity in *P. koreensis* S150.

## 2 MATERIALS AND METHODS

### 2.1 Isolation and identification of fluorescent *Pseudomonas* spp

Soil samples were collected from the tobacco rhizosphere at the Yanhe Base of Yunnan Tobacco Research Institute, China. Two grams of soil sample was suspended in 8 ml of sterile distilled water, and 250 µl of the soil suspension was spread onto King's B plates (King, Ward, & Raney, 1954). After 72 hr at 30°C, individual colonies with fluorescence at UV 365 nm were transferred and streaked onto new plates for confirmation.

Cell morphology was observed with a transmission electron microscope (Hitachi‐H600, Japan) after staining negatively with 1% (w/v) phosphotungstic acid. Phenotypic profiles of bacterial isolates were analyzed using GEN III MicroPlates™ (Biolog), which included 94 phenotypic tests, 71 carbon source utilization assays, and 23 chemical sensitivity assays, according to the manufacturer's protocol. The utilization pattern was indicated by the reduction of tetrazolium salt, which is a redox indicator dye that changes from colorless to purple in the well, and then it is monitored as absorbance with an OmniLog^®^ Incubator/Reader (Biolog) at 590 nm. The data were collected using OmniLog^®^ MiroArray™ Data Collection Software 1.2 (Biolog). This assay was repeated twice.

For 16S rDNA determination, the bacterial chromosomal DNA was prepared following a standard procedure (Sambrook & Russell, 2001). Oligonucleotide primers 27F (5'‐GAGAGTTTGATCCTGGCTCAG‐3') and 1494R (5'‐CTACGGCTACCTTGTTACGA‐3') (Weisburg, Barns, Pelletier, & Lane, 1991) were used for 16S rDNA amplification. PCR reactions and 16S rDNA cloning were carried out as described previously (Lei, Xia, Liu, & Wei, 2015). The positive clone was sequenced at GENEWIZ Ltd., China. The 16S rDNA (1,284 bp), *gyrB* (508 bp), *ropB* (443 bp), and *ropD* (430 bp) gene sequences were extracted from the PseudoMLSA Database (http://microbiologia.uib.es/bioinformatica/) (Bennasar, Mulet, Lalucat, & García‐Valdés, 2010). The accession numbers of the genes used in this study are listed in Table A1. Multiple alignments of nucleotide gene sequences were created using ClustalX and MEGA X software (Kumar, Stecher, Li, Knyaz, & Tamura, 2018). The neighbor‐joining method with the p‐distance method was used to construct phylogenetic trees (Kumar et al., 2018). The robustness of individual branches was estimated by bootstrapping with 1,000 replications.

### 2.2 Assays for antagonistic capacity against plant pathogens

The antagonism of strain S150 against *Phytophthora nicotianae* and *Rhizoctonia solani* on PDA (Potato Dextrose Agar) was performed as follows. Fresh mycelial disks (diameter, 5 mm) of the fungi were inoculated onto the center of fresh PDA plates (diameter, 90 mm), and 5 µl of saturated strain S150 culture was dotted around the inocula at a distance of 30 mm. The plates were incubated at 25°C for 3–4 days and antibiosis ability was determined by measuring the inhibitory zones.

To test the antagonistic capacity to phytobacteria, 5 ml of saturated *Ralstonia solanacearum* and 5 ml of *Xanthomonas oryzae* culture were mixed with 50°C LB media, respectively, and poured onto plates. Five µl of saturated strain S150 culture was placed on the plate center and incubated at 28°C. After 2–3 days, we measured the inhibitory zones and took photographs. All the antagonism assays were performed in triplicate at least twice independently.

### 2.3 Plant growth‐promoting assays

The strain was tested for important traits that promote plant growth. The assays for phosphate solubilization, potassium solubilization, and production of indole‐3‐acetic acid (IAA), ammonia, siderophores, protease, amylase, and cellulase were based on a previous report (Cappuccino & Sherman, 1996). For each trait, the experiments were conducted in triplicate, and the experiment was repeated two times.

*Arabidopsis thaliana* Col‐0 seeds were surface‐sterilized by soaking in 70% ethanol for 5 min followed by rinsing in sterile distilled water and sown on square Petri dishes with agar‐solidified Murashige and Skoog (MS) medium supplemented with 0.5% sucrose as described (Zamioudis, Mastranesti, Dhonukshe, Blilou, & Pieterse, 2013). The seeds on MS agar were stratified for 2 days at 4°C and subsequently incubated in a vertical position in a plant growth chamber at 21°C with a 16/8 hr photoperiod. After 4 days of growth, 250 µl of a bacterial suspension in 10 mM MgSO_4_ that contained 10^8^ cfu/ml was applied to the agar medium 5 cm below the roots of the seedlings. The same volume of MgSO_4_ (10 mM) was applied as a mock treatment. After 8 days, lateral root numbers and fresh weight were determined. The experiments were repeated three times.

### 2.4 Genome sequencing, annotation, and comparison

Genomic DNA was extracted from fresh bacterial culture according to standard procedure (Sambrook & Russell, 2001). Purified DNA was sent to GENEWIZ Ltd., China for library construction. The complete genome was sequenced in a PacBio RSII instrument, then the PacBio reads were filtered and assembled using HGAP (Hierarchical Genome Assembly Process) with default parameters (github.com/PacificBiosciences/Bioinformatics‐Training/wiki/HGAP). The coding genes were annotated using the National Center for Biotechnology Information (NCBI) nr database using the Diamond protein aligner (Buchfink, Xie, & Huson, 2015; NCBI Resource Coordinators, 2018). Then, the functions of genes were annotated using the GO (Gene Ontology) database (Gene Ontology Consortium, 2004), and the pathways were annotated using the KEGG (Kyoto Encyclopedia of Genes and Genomes) database (https://www.genome.jp/kegg/pathway) (Kanehisa & Goto, 2000). The comparative analysis of the chromosomes between strain S150 and other *Pseudomonas* species was performed using GenomeMatcher software (http://www.ige.tohoku.ac.jp/joho/gmProject/gmhomeJP.html) and the bl2seq program, which is embedded in the bundled application (Ohtsubo, Ikeda‐Ohtsubo, Nagata, & Tsuda, 2008). BLAST Ring Image Generator (BRIG) was used for genome comparison (Alikhan, Petty, Ben Zakour, & Beatson, 2011). The circular genomic map was constructed with BLAST+ with standard default parameters. Secondary metabolite biosynthetic gene clusters (BGCs) were predicted by antiSMASH 5.0, a web‐based analysis platform (http://antismash.secondarymetabolites.org/) (Blin et al., 2019). BGCs were then clustered into groups based on protein sequence similarity using BiG‐SCAPE with default parameters and a distance cut‐off score of 0.3 (Navarro‐Muñoz et al., 2020). The Bacterial Pan Genome Analysis (BPGA) pipeline was used for the pan‐genome and core genome analyses (Chaudhari, Gupta, & Dutta, 2016). The cut‐off value of sequence identity was set up to 50% to obtain the pan and core genomes. Venn diagrams were drawn using OrthoVenn2, which is a web server for the comparison and analysis of whole‐genome orthologous clusters (Xu et al., 2019). Orthologous CDSs in the four genomes were defined after comparing all‐against‐all using DIAMOND (v0.9.24) (Buchfink et al., 2015) and processed by the OrthoMCL pipeline using default settings (Xu et al., 2019).

### 2.5 Gene mutation and complementation

A 7.0 kb fragment that covered the entire gene of *lokA* and the promoter region was amplified from the genome of strain S150 using primers LokA‐f (5'‐CGC AAG CTT CTC TCC TTG AAA CTG‐3') and LokA‐r (5'‐CAG TCT AGA GTC CGT GCC TGC TGT G‐3') and high‐fidelity DNA polymerase. After it was digested by *Hin*d III and *Xba* I, the fragment was ligated with pK18mobsacB, which was also digested by the same two endonucleases. The 6.4 kb DNA sequence of the lokA gene has four *Cla* I sites. The positive clone was then digested by *Cla* I to eliminate a 5.7 kb fragment of the *lokA* gene. The final construct was designated as pK18‐LokA, which was then introduced into strain S150 to make a *lokA* deletion mutant, and this was followed by two recombination steps (Liu, Zhang, Zhang, Liu, & Wei, 2015). The 7.0 kb fragment that was digested by *Hin*d III and *Xba* I was inserted at the same sites on the shuttle vector pBBR1MCS‐2 to generate the complementation plasmid pBBR‐LokA.

## 3 RESULTS

### 3.1 In vitro antimicrobial activity

**FIGURE 1:**
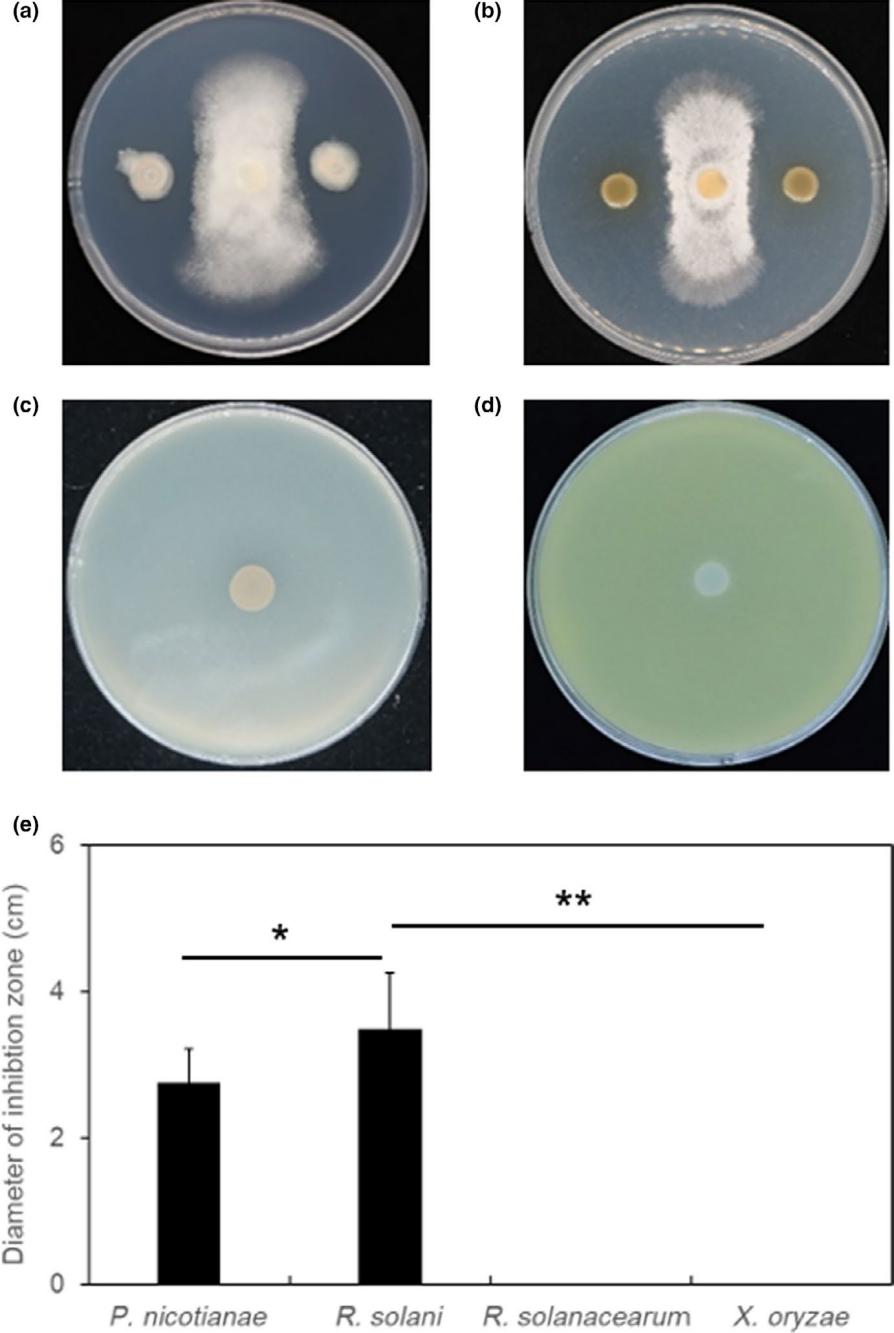
In vitro antimicrobial activity of strain S150 against different phytopathogens in the dual culture. (a) *Phytophthora nicotianae*, (b) *Rhizoctonia solani*, (c) *Ralstonia solanacearum*, (d) *Xanthomonas oryzae*. Antimicrobial activity was estimated by measuring the diameter (cm) of the clear zone of growth inhibition and shown as in (e). Results are expressed as the mean and standard deviation (Tukey's HSD test; *p* < 0.01). All the experiments were repeated three times with similar results

Using King's B selective medium, we isolated 65 fluorescent bacterial strains from the tobacco rhizosphere. All isolates were tested for antagonistic activity against plant pathogens. Among the isolates, one strain named S150 showed specific antagonistic ability against plant pathogenic fungi (*Phytophthora nicotianae* and *Rhizoctonia solani*) rather than against phytobacteria (*Ralstonia solanacearum* and *Xanthomonas oryzae*) (Figure 1). *P. nicotianae* and *R. solani* are two of the most devastating plant fungal pathogens that cause root rot diseases in various crops (Harris & Nelson, 1999). It shows the potential to exploit biocontrol agents for the control of soil‐borne fungal diseases specifically.

### 3.2 Plant growth‐promoting activity of strain S150

**FIGURE 2:**
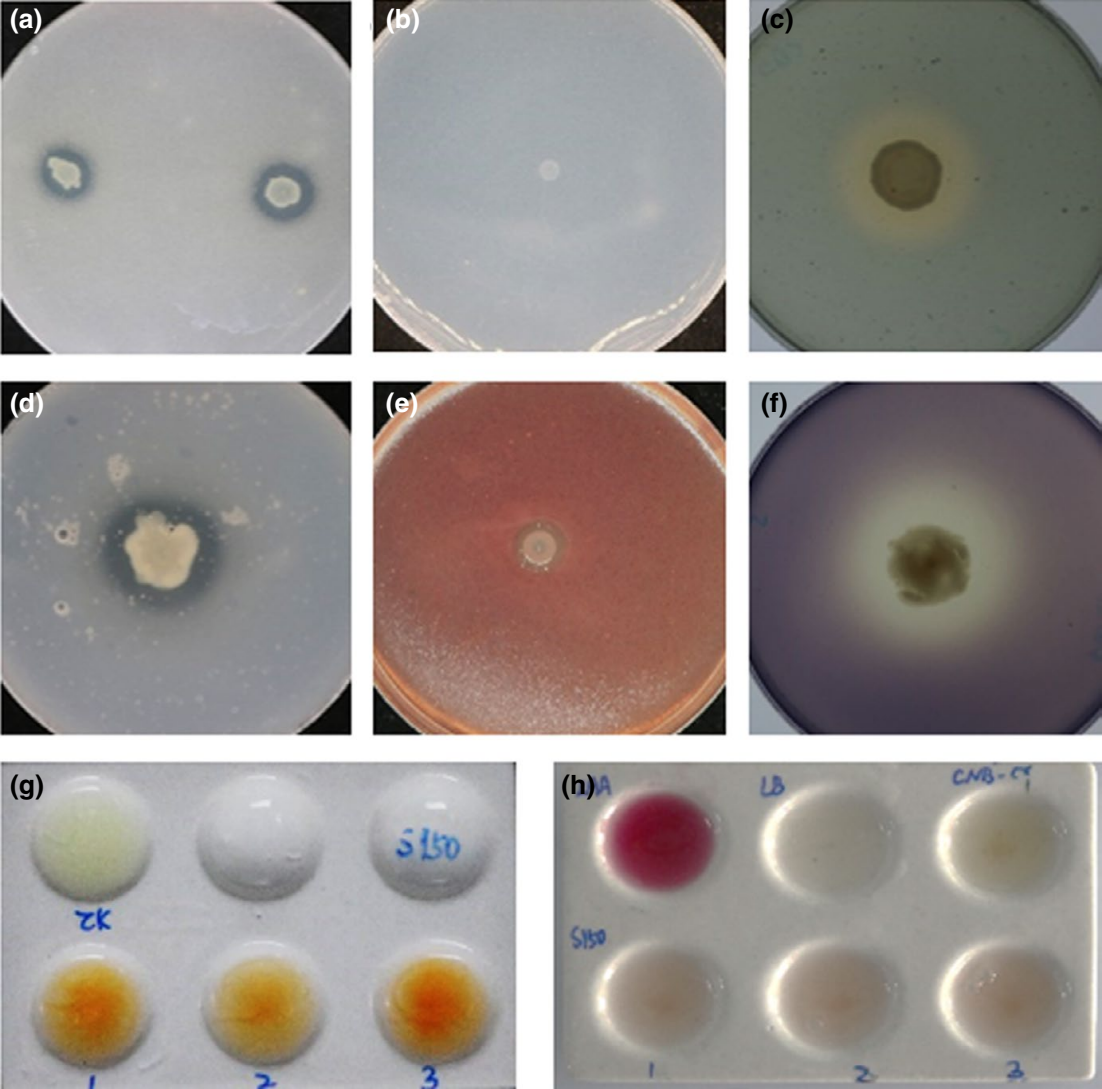
In vitro test of plant growth‐promoting traits of strain S150. The transparent zone around the colonies could be visualized from (a) phosphate solubilization, (c) siderophore production, (d) protease production, (e) cellulase production, and (f) amylase production. No potassium‐solubilizing ability was detected from a (b) potassium solubilization assay. (g) Ammonia production is shown from the visualized brown/yellow color‐treated with strain S150 (lower three replicates) compared with the upper left control. (h) Strain S150 is indole‐3‐acetic acid (IAA) negative compared with the positive control at the upper left. All the experiments were repeated three times with similar results

Phosphorus is a very important nutrient for plant growth (Richardson, Barea, McNeill, & Prigent‐Combaret, 2009). Thus, solubilizing phosphate is an important feature of PGPR to enhance nutrition through an increase in phosphorus uptake by plants (Richardson et al., 2009). In this study, the formation of a clear solubilization zone around the colonies suggested that strain S150 was capable of solubilizing phosphate (Figure 2a). On the Aleksandrov plates, strain S150 did not show convex, slimy, elastic, and translucent colonies, which indicated that the strain had no potassium‐solubilizing ability (Figure 2b). An orange halo developed around the colonies on the blue Chrome Azurol S (CAS) agar medium, which signified its ability to produce siderophore (Figure 2c). Siderophores are low molecular‐mass compounds that are utilized by bacteria as iron (Fe) chelating agents (Saha, Saha, Donofrio, & Bestervelt, 2012). Siderophore‐producing PGPR can prevent the proliferation of pathogens by sequestering Fe^3+^ in the plant rhizosphere (Schiessl, Janssen, Kraemer, McNeill, & Ackermann, 2017). Enzymatic assays showed that strain S150 produced hydrolytic enzymes, such as protease (Figure 2d), cellulase (Figure 2e), and amylase (Figure 2f). Strain S150 also displayed positive results for ammonia production (Figure 2g), but not for IAA production, the most common plant hormone that regulates various aspects of plant growth and development (Gray, 2004) (Figure 2h).

**FIGURE 3:**
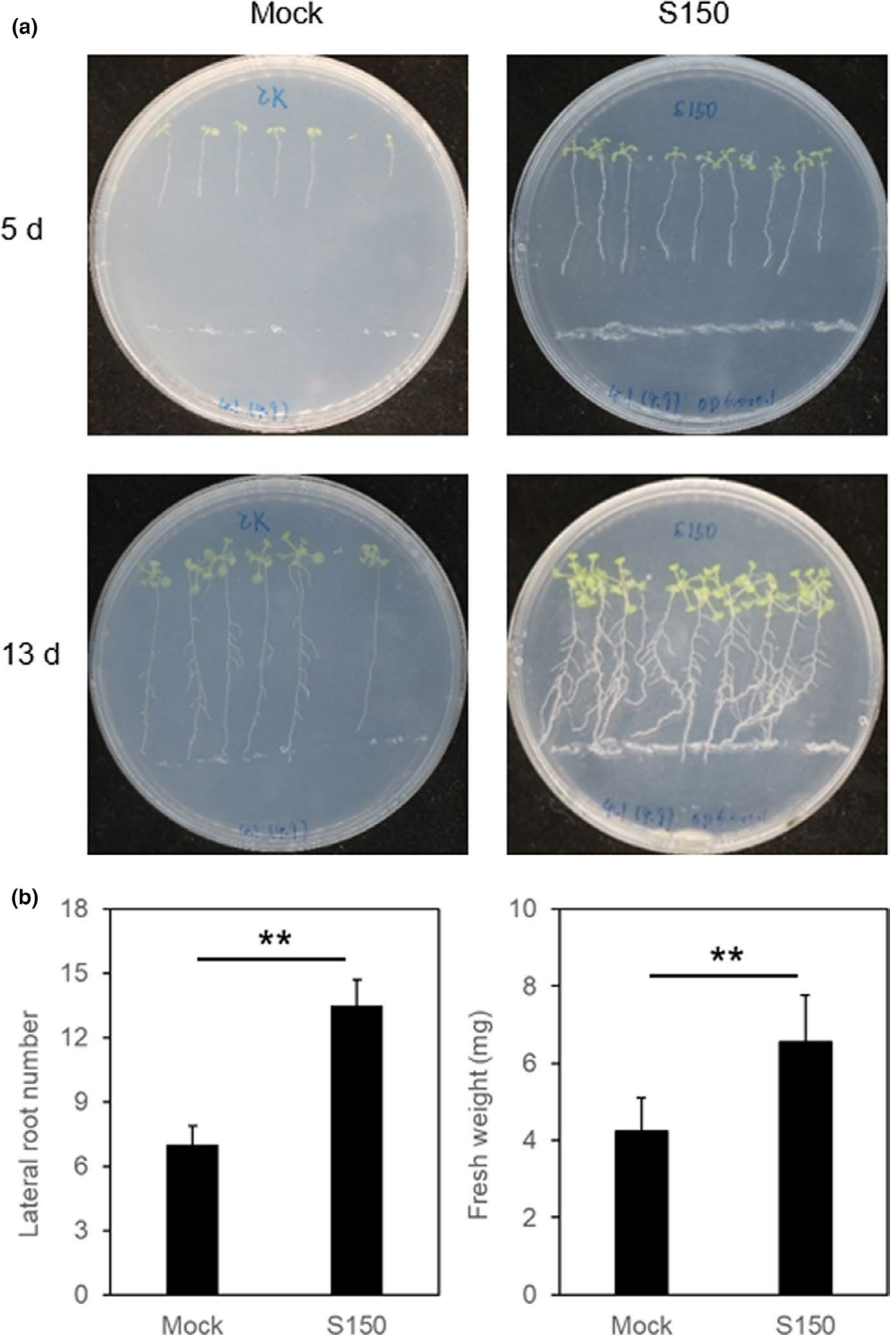
Effects of strain S150 on plant growth and root system architecture of *Arabidopsis* Col‐0 seedlings. (a) Seedlings growing on control plates and plates that contained strain S150. (b) Shoot biomass production measured after 13 d of cocultivation with mock and strain S150. Stars indicate statistically significant differences (Tukey's HSD test; *p* < 0.01). The experiment was repeated twice with similar results

The plant growth‐promoting effect of strain S150 on *Arabidopsis* ecotype Columbia (Col‐0) seedlings was determined. Root length, lateral number, and plant biomass production were determined. Interestingly, in the first few days of cocultivation, strain S150 promoted primary root elongation significantly compared to the mock control (Figure 3a). Over time, root elongation slowed, and the number of lateral roots and root hairs increased (Figure 3a). At 13 dpi (Days post‐inoculation), we measured increases of 2‐fold in the lateral root number and 1.5‐fold in the shoot fresh weight of seedlings that grew in the presence of strain S150 (Figure 3b). These results indicated that S150 was an efficient PGPR capable of stimulating plant biomass production. However, it is distinctly different from the model PGPR strain WCS417, which promotes lateral root formation and biomass production but inhibits primary root length (Zamioudis et al., 2013). These results suggested that S150 produced specific diffusible and/or volatile compounds to promote plant growth.

### 3.3 Identification of strain S150

**FIGURE 4:**
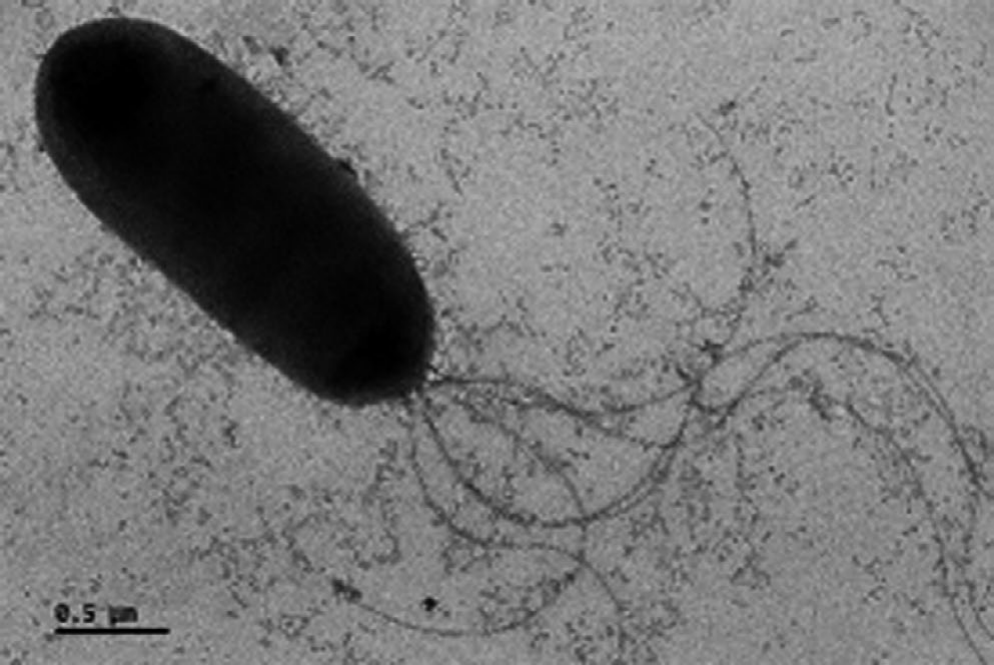
Electron micrograph of strain S150 cells

The colonies of strain S150 on King's B media were circular, convex, and yellow. The cells of strain S150 were rod‐shaped, 0.5–1.1 μm in diameter, and 1.4–4.2 μm long. More than one flagellum at one polar was determined by a transmission electron microscope (Figure 4). According to the results of the GEN III MicroPlates™ assay, strain S150 had different utilization patterns for carbohydrates, amino acids, carboxylic acids, and their derivatives (Table A2). Briefly, the assay showed that strain S150 utilized d‐fructose, α‐d‐glucose, citric acid, dextrin, d‐mannitol, d‐mannose, d‐trehalose, d‐sorbitol, l‐glutamic acid, etc. But it did not assimilate maltose, l‐pyroglutamic acid, and p‐hydroxy‐phenylacetic acid. All these characters are very close to the type strain LMG 21318^T^ of *Pseudomonas koreensis* (Kwon et al., 2003).

**FIGURE 5:**
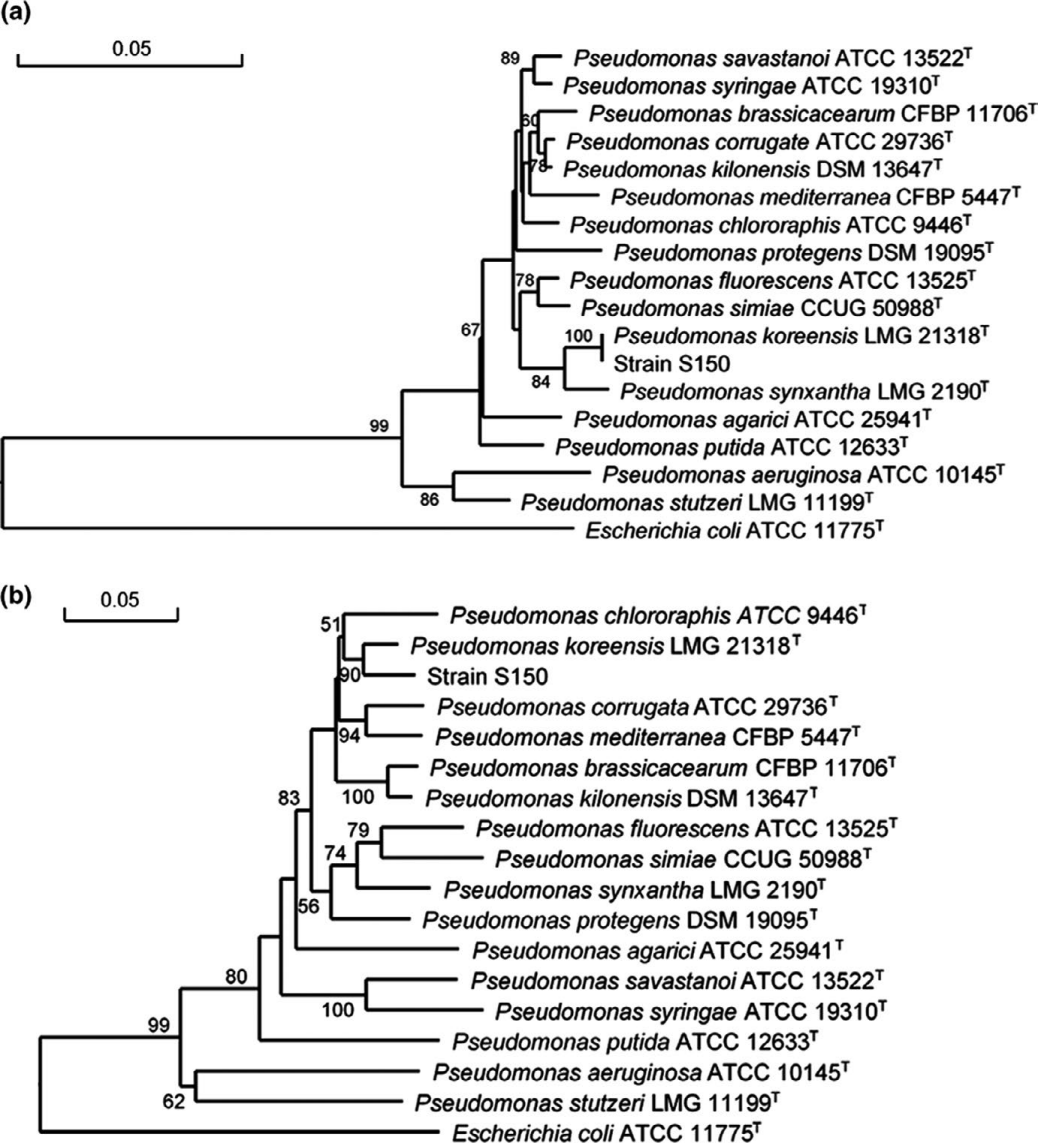
Phylogenetic trees showing the relationship of *P. koreensis* S150 with representative type strains of *Pseudomonas* spp. The trees are based on the alignment of the 16s rDNA sequences (a) and concatenated sequences of four core housekeeping genes (16S rRNA, *gyrB*, *rpoB*, *rpoD*) (b) of the strains. Bootstrap values from 1000 replicates are indicated at the nodes

The complete genome of strain S150 was sequenced, annotated, and deposited in NCBI (accession no. CP038207). The 16S rDNA sequence of strain S150 was very similar to that of *P. koreensis*. To obtain a comprehensive overview of the taxonomic position of strain S150, we extracted *gyrB*, *ropB*, and *ropD* gene sequences from the genome and compared them with the corresponding genes of representative *Pseudomonas* type species. Phylogenetic trees were constructed based on 16S rDNA sequences (Figure 5a) and multi‐locus sequence analysis (MLSA) (Figure 5b) of the concatenated sequences of four housekeeping genes. Both trees showed that strain S150 was related most closely to the *P. koreensis* type strain LMG 21318^T^. In conclusion, sequence analysis (16S rDNA and MLSA), along with morphological, physiological, and biochemical characteristics, revealed that strain S150 belonged to *P. koreensis*. A culture of S150 was deposited in the China General Microorganism Culture Collection (AS1828).

### 3.4 General genome characteristics

**FIGURE 6:**
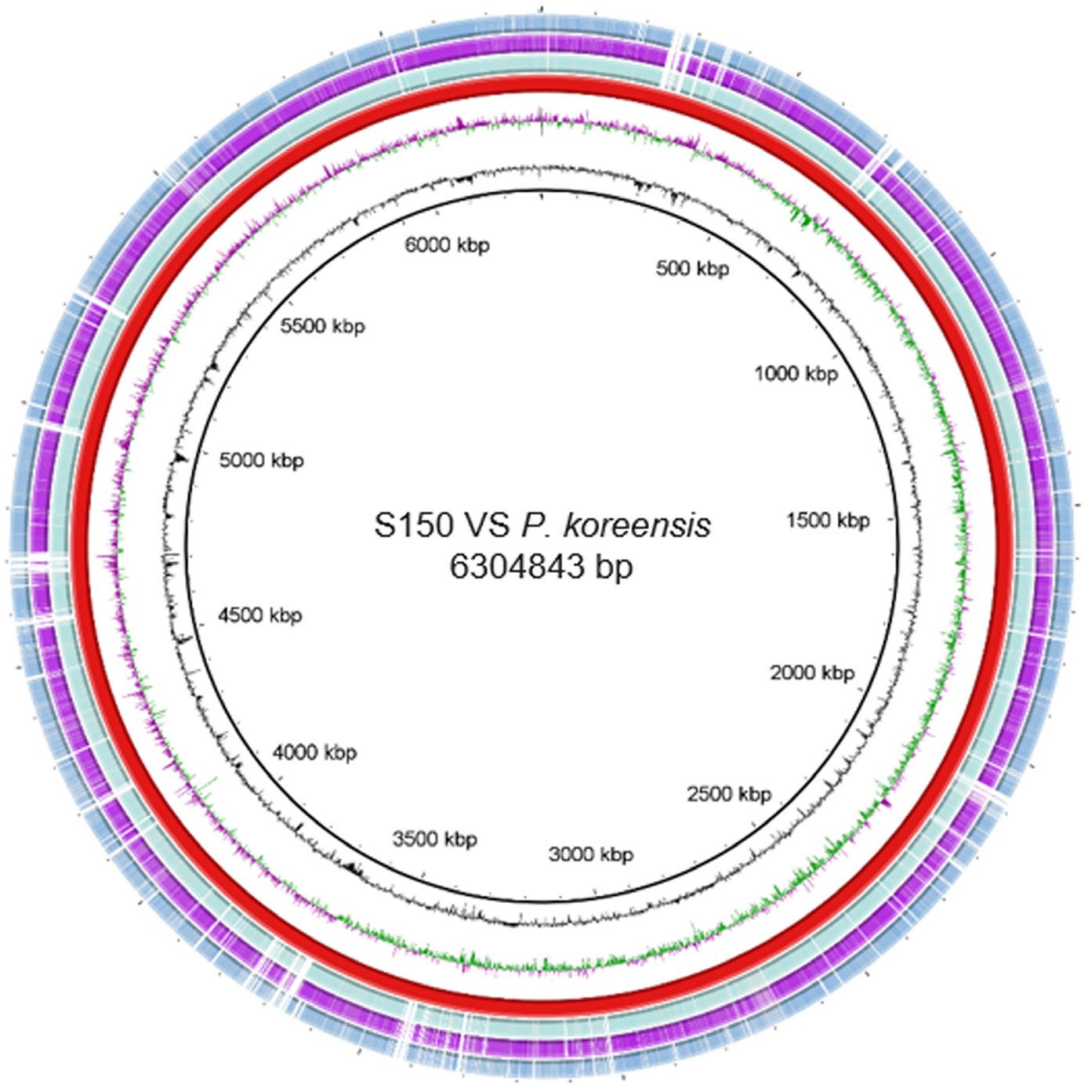
Circular map of the chromosome of strain S150 and other *P. koreensis* genomes. The complete genome of *P. koreensis* strain D26 was taken as a reference genome. The tracks from the inside to outside: GC Content, GC Skew, *P. koreensis* D26 (red), *P. koreensis* BS3658 (light blue), *P. koreensis* CRS05‐R5 (purple), and *P. koreensis* S150 (blue)

To obtain comprehensive information, the complete genome of strain S150 was sequenced at GENEWIZ Ltd., China (Table 1). The size of the genome was 6,304,843 bp in a single circular chromosome with an average G + C content of 61.1% (Figure 6). A total of 5,773 coding sequences (CDSs) was predicted, which included 5,454 protein‐encoding sequences based on similarity searches and experimental evidence, and this accounted for 87.7% of the total genome. The average CDS length was 973 bp. Also, 319 pseudogenes that were due to a missing N‐ and/or C‐terminus or a frameshift mutation, 71 tRNA, 19 rRNAs, and 4 non‐coding RNA genes were predicted to occur on the chromosome. No plasmid was found in strain S150.

**TABLE 1:** General features of strain S150 genome

Features	Chromosome
Size (bp)	6,304,843
G + C content (%)	61.1
Number of total CDSs	5,773
Number of genes	5,454
Pseudogenes	319
tRNAs	71
rRNA genes	19
ncRNAs	4
Contigs	1
Total CDSs size (bp)	5,529,288
Coding %	87.7
Average CDS length (nt)	972.6

**FIGURE 7:**
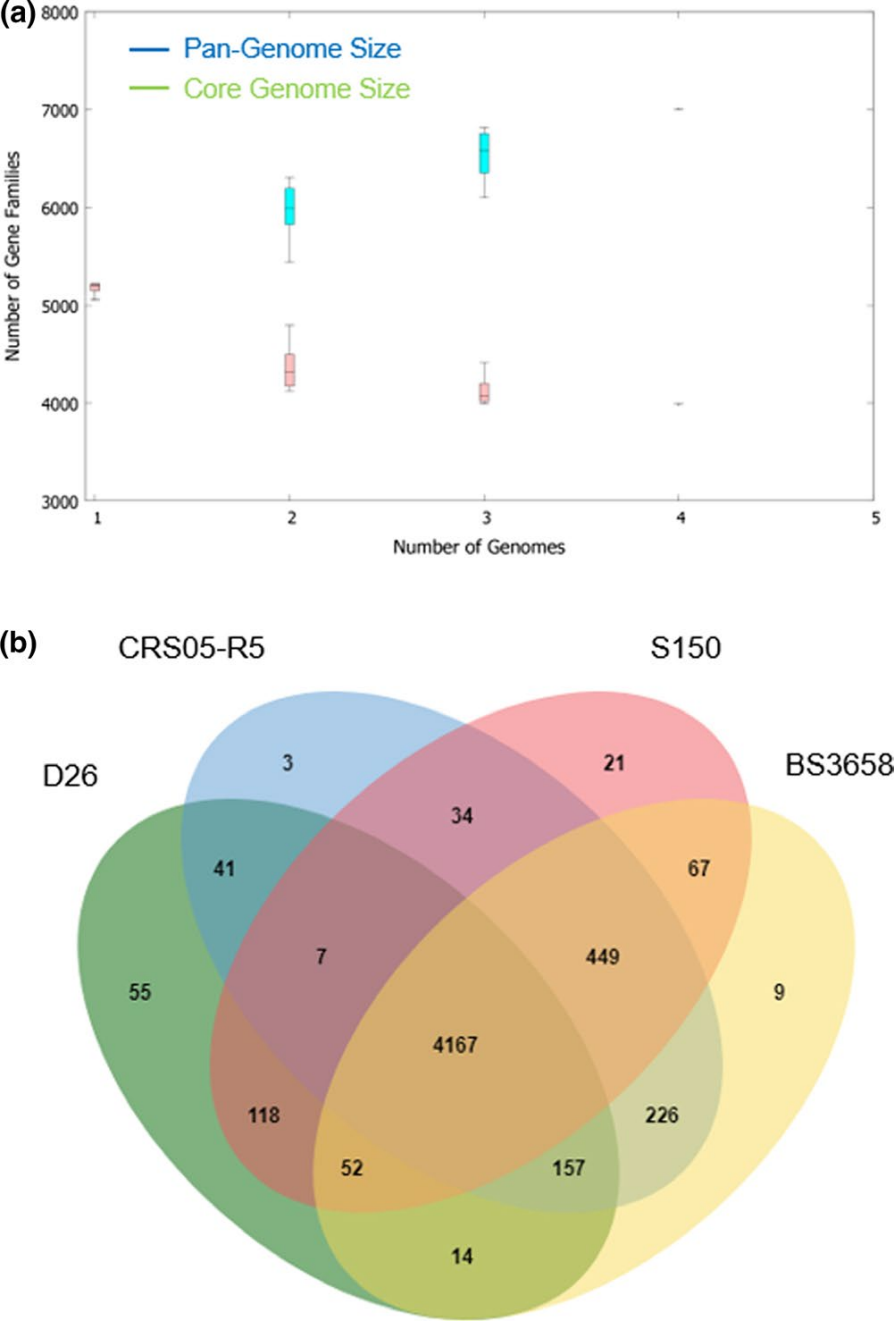
Core and pan‐genome analysis of the four strains *P. koreensis*. (a) An estimate of core genome size and pangenome size using BPGA. (b) Venn diagram of orthologous clusters generated by OrthoVenn

A pan‐genome for the strain S150 and three fully sequenced *P. koreensis* strains D16 (accession no. CP014947), CRS05‐R5 (accession no. CP015852), and BS3658 (LT629687) was determined by BPGA, comparing the translated gene set, followed by clustering of gene families and core conserved genes in the total pan‐genome. The total pan‐genome for the 4 compared *P. koreensis* strains encompasses 9,320 genes which constitute 7,005 groups of orthologous genes (Figure 7a). Of these, 3,986 (42.8% of total genes) are core conserved genes across all 4 strain genomes. Further comparison and annotation of orthologous gene clusters among the 4 strains using OrthoVenn indicated up to 4,167 homologous gene clusters were shared among these four genomes, and 21 orthologous clusters were present only in the strain S150 genome (Figure 7b). The unique clusters that existed in S150 involved genes that functioned in zinc ion binding, oxidoreductase activity, regulation of transcription, nuclease binding, and negative regulation of secondary metabolite biosynthetic processes (Table A3). Further investigation to understand the features of these unique genes in S150 is warranted.

### 3.5 Gene prediction involved in secondary metabolites

**FIGURE 8:**
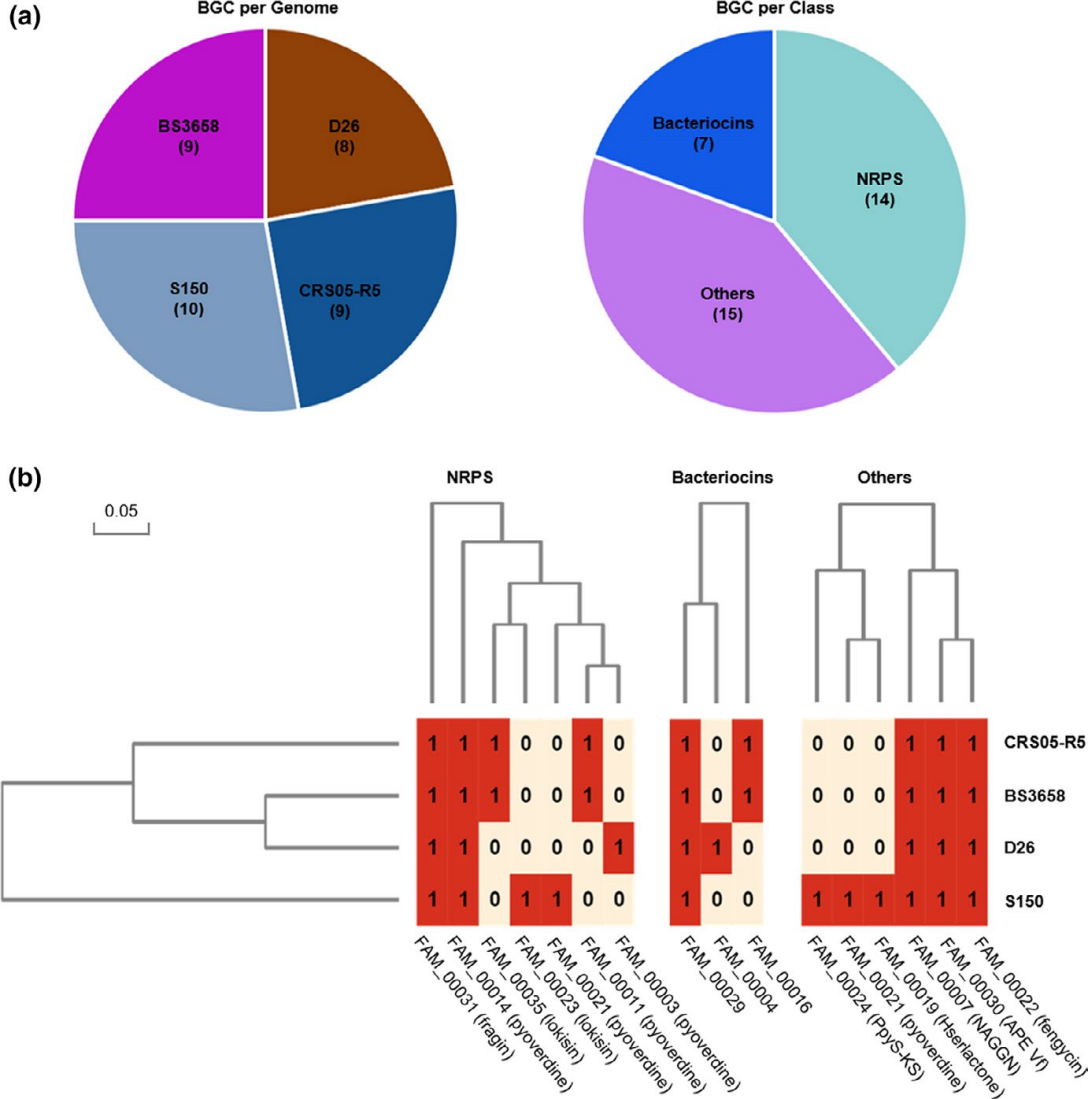
Comparative analysis of BGCs detected in *P. koreensis* S150 against three other strains. (a) BGCs prediction and classification by antiSMASH. Brackets contain the BGC numbers. (b) Analysis of gene cluster families using BiG‐SCAPE based on the distance matrix. 0 represents absence and 1 represents presence

Due to the antagonistic and plant growth‐promoting capacity, we focused on secondary metabolite biosynthetic genes in strain S150. Based on the antiSMASH platform, 10 BGCs for secondary metabolites were predicted for the genome of *P. koreensis* S150 (Figure 8a). No polyketide (PKS) biosynthetic genes such as DAPG, phenazine, or pyrrolnitrin were present on the chromosome. To find the specific metabolites, we also did a comparative analysis of the BGCs for secondary metabolites between strain S150 and the other three sequenced *P. koreensis* strains D26, CRS05‐R5, and BS368. All four strains have different BGC profiles in which strain S150 has the largest BGC numbers while D26 has fewest (Figure 8a). The BGCs were clustered into three classes based on the predicted chemical features including NRPS, bacteriocins, and others, which contains 14, 7, and 15 BGCs, respectively (Figure 7a). A further clustering in class using BiG‐SCAPE based on sequence similarity grouped them into gene cluster families (GCFs), each of which contained BGCs across the selected bacterial strains (Figure 8b). As distinct from the other three strains, S150 had four extra GCFs. One unique 20.5 kb GCF with 18 genes was predicted as hserlactone (cluster coding for homoserine lactone; FAM_00019), and another 21.0 kb GCF with 18 genes was predicted to synthesize PpyS‐KS (FAM_00024). However, no functional characterization was reported regarding these two GCFs and their products. Alternatively, a large 76.3 kb fragment that included three NRPS‐encoding genes was predicted to produce lokisin (Table 2), a member of the amphisin group of CLPs that consisted of 11 AAs (Sørensen et al., 2002). The lokisin gene clusters were present in strains CRS05‐R5 and BS3658, but not in strain D26, which suggested that we needed to determine the function of the gene cluster of lokisin.

**TABLE 2:** Annotation of the gene cluster for lokisin biosynthesis

Gene ID	Location	Strand	Identity to COR10 (%)	Annotation
E3Z29_14735	3213281–3214298	+	99	Sulfonate ABC transporter substrate‐binding protein
E3Z29_14740	3214425–3214920	+	90	Hypothetical protein
E3Z29_14745	3214989–3215469	−	99	Copper chaperone PCu(A)C
E3Z29_14750	3215468–3216074	−	100	SCO family protein
E3Z29_14755	3216230–3217634	−	98	Efflux transporter outer membrane subunit
E3Z29_14760	3217721–3218516	−	99	LuxR family transcriptional regulator
E3Z29_14765	3219004–3225418	+	97	Amino acid adenylation domain‐containing protein
E3Z29_14770	3225599–3238614	+	99	Amino acid adenylation domain‐containing protein
E3Z29_14775	3238610–3256454	+	90	Non‐ribosomal peptide synthetase
E3Z29_14780	3256520–3257669	+	100	Macrolide transporter subunit MacA
E3Z29_14785	3257671–3259630	+	100	MacB family efflux pump subunit
E3Z29_14790	3260521–3260893	+	98	Hypothetical protein
E3Z29_14795	3261031–3261391	+	94	Hypothetical protein

### 3.6 Lokisin‐encoding genes were associated with anti‐fungal capacity

**FIGURE 9:**
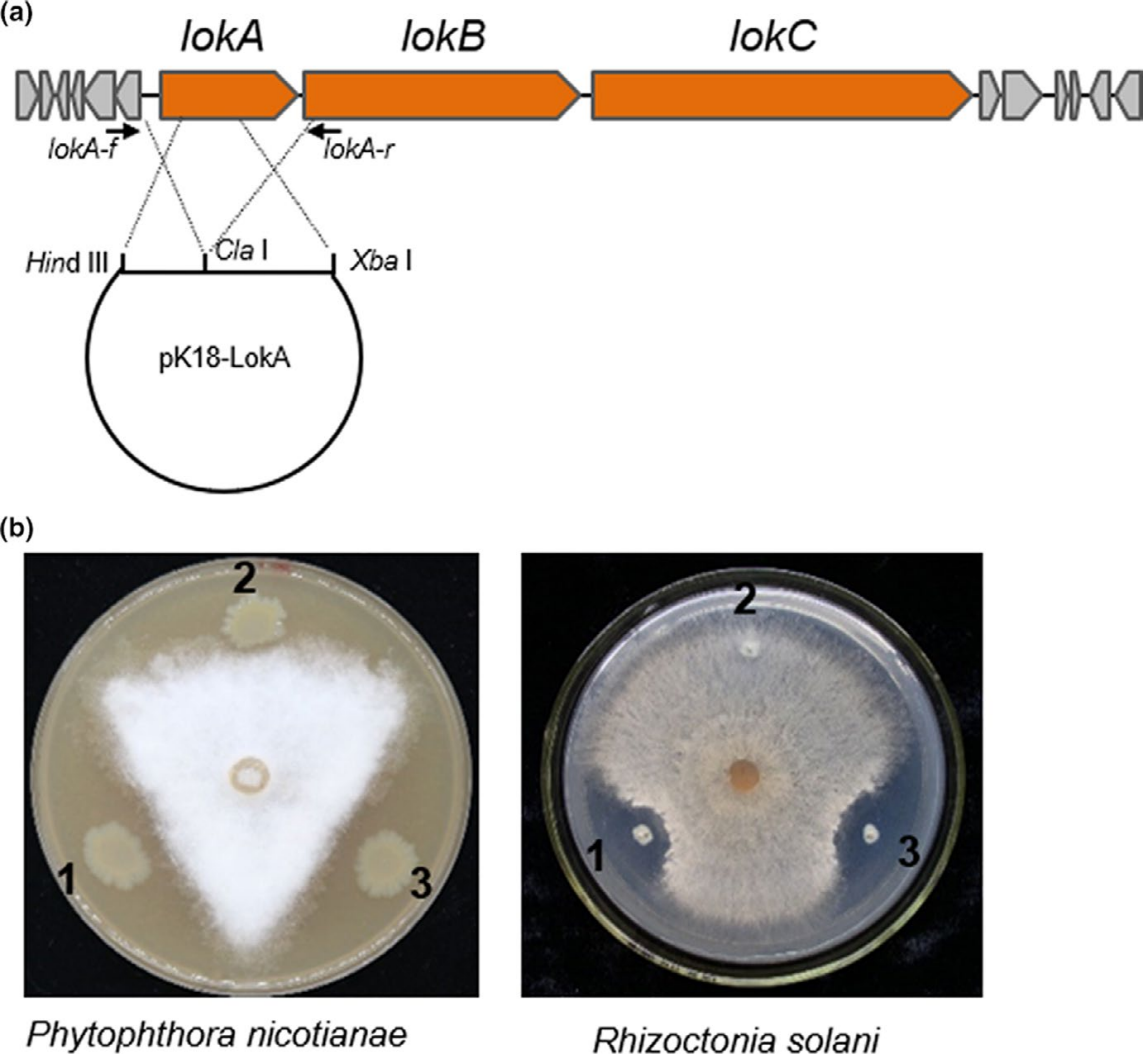
The lokisin gene cluster and mutation analysis of *lokA* gene. (a) Schematic and mutation of the lokisin gene cluster. The arrows represent the primers. The dotted lines represent double crossover recombination to generate a *lokA* mutant. (b) Antagonistic assay to show that the *lokA* mutant has reduced activity against *P. nicotianae* specifically and a complete lack of inhibition against *R. solani*. 1, wild type strain S150; 2, *lokA* mutant; 3, *lokA* complementation strain

Lokisin was first isolated from *Pseudomonas* sp. DSS41, but the genome sequence is still not available. Recently, a 50.1 kb fragment that harbored a lokisin gene cluster of *Pseudomonas* sp. COR10 was published and deposited in GenBank with accession number MK534107 (Omoboye et al., 2019). A Blast and alignment analysis showed that all of the proteins encoded by the 13 core genes of lokisin exhibited >90% identity between strains S150 and COR10. The three major NRPS genes *lokA* (E3Z29_14765), *lokB* (E3Z29_14770), and *lokC* (E3Z29_14775) encoded two, four, and five AAs of the lokisin structure, respectively; The amino acid sequences of LokA, LokB, and LokC had 97%, 99%, and 100% identities, respectively, to those in strain COR10, which served as a reliable clue to elucidate lokisin production in *P. koreensis* S150. The *lokA* gene encoded the smallest NRPS with 2,317 AA. We then made a deletion mutant of the *lokA* gene and tested its antifungal activity (Figure 9a). The *lokA* mutant failed to antagonize against *R. solani* and reduced sharply antagonistic capacity against *P. nicotianae* in a dual culture assay (Figure 9b). The full gene complementation of *lokA* restored the antifungal level to the same level as wild type strain S150 (Figure 8b). These results indicated that lokisin that was synthesized by the NRPS cluster played an important role in antagonism against plant fungal pathogens.

## 4 DISCUSSION

Since its recognition as a new species, some *P. koreensis* strains were reported as being PGPR and bioremediation bacteria (Babu, Shea, Sudhakar, Jung, & Oh, 2015; Hultberg et al., 2010; Jeong, Rha, Kim, & Lee, 2018; Lin et al., 2016; Lozano, Bravo, & Handelsman, 2017; Rafikova, Korshunova, Minnebaev, Chetverikov, & Loginov, 2016; Srivastava et al., 2019). In this study, we reported a newly isolated *P. koreensis* strain S150, which antagonized plant pathogens, solubilized phosphates, produced siderophores and enzymes, and promoted plant growth. To date, 16 strains of the species *P. koreensis* have been sequenced, but only three strains have whole genome sequences (https://www.ncbi.nlm.nih.gov/genome/genomes/44404). However, the genome sequence of *P. koreensis* P19E3, which is one of the three completely sequenced strains, was noted on the website as being contaminated. Therefore, *P. koreensis* S150 is the third strain that is being fully sequenced successfully, which would enrich the genome information for this species. From the genome analysis, we saw that strain S150 synthesized two siderophores (FAM_00014 and FAM_00021) (Figure 8b). This phenomenon was also found with *P. entomophila* L48, which harbored two synthesis gene clusters of pyoverdine (Matthijs et al., 2009). The ability of bacteria to produce multiple siderophores offers protection against exploitation from non‐producers with incompatible receptors in natural *Pseudomonas* communities (Butaitė, Baumgartner, Wyder, & Kümmerli, 2017; Matthijs et al., 2009). However, the precise structures of the siderophores in *P. koreensis* S150 need to be confirmed experimentally.

*Pseudomonas koreensis* S150 is a plant growth‐promoting strain, which increased the lateral root number and shoot fresh weight. PGPR strains possess a few IAA synthesis pathways to promote plant root development (Gross & Loper, 2009). A search of the sequenced genome for IAA pathway‐associated genes revealed the absence of orthologous genes in *P. koreensis* S150. However, a homologous gene *acdS* (E3Z29_13135) that encoded 1‐aminocyclopropane‐1‐carboxylate (ACC) deaminase was found in the genome. It had a length of 924 bp and encoded 307 AA, which possessed 97.7%, 96.7%, and 96.4% identities to ACC deaminases from *P. putida* (PHH43559), *P. fluorescens* (QBX40969), and *P. koreensis* (KAA8738874), respectively. The plant hormone ethylene is an important modulator for normal plant growth and development, and it was also associated with biotic and abiotic stress (Abeles, Morgan, & Saltveit, 1992). Many PGPR strains can hydrolyze the ethylene precursor ACC into ammonia and α‐ketobutyrate with AcdS activity (Heydarian et al., 2016; Onofre‐Lemus, Hernández‐Lucas, Girard, & Caballero‐Mellado, 2009). It has thus been proposed that AcdS activity of PGPR strains could decrease the level of ethylene in roots, which in turn may promote root development and enhance plant tolerance against pathogenic infections and antagonize pathogens (Glick, 2014). Further functional annotation of the genome revealed that strain S150 harbored the genes responsible for ammonia assimilation through the glutamine synthetase (GS)‐glutamate synthase (GOGAT) pathway using glutamine synthetase (GlnA, WP_003220788). Hence, more studies are needed to identify precisely the prevailing molecular mechanisms behind such activity that would benefit plant growth and be useful for biological control.

Lokisin is a rare amphisin‐type CLP, which inhibited fungal growth (Hultberg et al., 2010; Sørensen et al., 2002). Nielsen reported that the lokisin‐producing strains only possessed 6% of a pool of 600 CLP‐positive fluorescent *Pseudomonas* (Nielsen et al., 2002). The relatively large lipopeptide lokisin was more antagonistic toward plant pathogenic fungi than other CLPs (Nielsen et al., 2002). A more recent study showed that lokisin which was isolated from *Pseudomonas* sp. COR10 controlled rice blast disease caused by *Magnaporthe oryzae* by induced systemic resistance and direct antagonism against the pathogen (Omoboye et al., 2019). However, only a 50.3 kb fragment from strain COR10 that harbored the entire gene cluster of lokisin had been published before this study (Omoboye et al., 2019). The gene products in the cluster from the genome of *P. koreensis* S150 have very high identities (>90%) to those found from strain COR10, which enhances the possibility of production of lokisin in strain S150. The comparative in silico and antiSMASH analyses showed the gene cluster product of strain S150 was congruent with the chemical structure of lokisin (Oni et al., 2019; Sørensen et al., 2002) although we did not do isolation and purification of lokisin. To determine the function of lokisin, we made a deletion mutant of the smallest NRPS gene *lokA* in strain S150. Antifungal activity was sharply reduced in the *lokA* mutant and was restored by gene complementation. This is the first report to verify the antifungal activity of lokisin through genetic mutation in bacteria. But it needs to be pointed out that the *lokA* mutant still has a slight antagonistic activity against *P. nicotianae* compared to a complete failure against *R. solani*. It suggests that lokisin might not the only secondary metabolite in *P. koreensis* S150 against *P. nicotianae‐*like plant pathogenic oomycete. Interestingly, a further BLASTp comparison showed the draft genomes of the other two *P. koreensis* strains CCUG 51519 (LokABC, F7R05_07640‐ F7R05_07650) and P19E3 (LokABC, PkP19E3_15525‐ PkP19E3_15535) contained a high identity (>90%) of the lokisin gene cluster besides strains CRS05‐R5 and BS3658 analyzed above. These results suggested that *P. koreensis* might be a rich species that produces lokisin‐like CLPs, which is somewhat consistent with a proposal that used CLP genes as a taxonomic designation (Omoboye et al., 2019; Oni et al., 2019). However, further studies would be conducted to determine the yield of lokisin in varied *P. koreensis* strains and the practical application in plant disease control.

## CONFLICT OF INTEREST

None declared.

## AUTHOR CONTRIBUTIONS

**Yilin Gu:** Investigation (equal); Methodology (equal). **Yi‐Nan Ma:** Investigation (equal); Methodology (equal). **Jing Wang:** Investigation (supporting); Methodology (supporting). **Zhenyuan Xia:** Investigation (equal); Methodology (equal); Project administration (equal); Supervision (equal); Writing‐review & editing (equal). **Hai‐Lei Wei:** Project administration (equal); Supervision (equal); Writing‐original draft (equal); Writing‐review & editing (equal).

## ETHICS STATEMENT

None required.

## Data Availability

The dataset generated for this study is available in GenBank CP014947.

## APPENDIX 1

**TABLE A1:** Accession numbers of the bacterial type strains used for phylogenetic analysis

Strains	Accession number			
	16S rRNA	*gyrB*	*rpoD*	*rpoB*
*Escherichia coli* ATCC 11775^T^	X80725.1	AB083821.1	CP033092.2	CP033092.2
*Pseudomonas aeruginosa* ATCC 10145^T^	NR_114471.1	HQ425720.1	VAOQ01004775.1	VAOQ01002313.1
*Pseudomonas agarici* ATCC 25941^T^	NR_115608.1	LN849826.1	AB039563.1	AJ717477.1
*Pseudomonas brassicacearum* CFBP 11706^T^	AJ293858.1	AM084675.1	AM084334.1	NZ_VZPJ01000019.1
*Pseudomonas chlororaphis* ATCC 9446^T^	NR_114474.1	FJ652718.1	D86036.1	FJ652691.1
*Pseudomonas corrugata* ATCC 29736^T^	LN849842.1	AB039460.1	AB039566.1	NZ_LIGR01000008.1
*Pseudomonas fluorescens* ATCC 13525^T^	NR_114476.1	QVNA01000002.1	NZ_QVNA01000030.1	AJ717451.1
*Pseudomonas kilonensis* DSM 13647^T^	NR_028929.1	AM084677.2	AM084336.1	NZ_LHVH01000019.1
*Pseudomonas koreensis* LMG 21318^T^	NR_025228.1	GU078442.1	FN554476.1	FN554737.1
*Pseudomonas mediterranea* CFBP 5447^T^	NR_028826.1	AM084678.2	AM084337.2	AUPB01000038.1
*Pseudomonas protegens* DSM 19095^T^	NR_114749.1	HE800482.1	DQ458677.1	HE800514.1
*Pseudomonas putida* ATCC 12633^T^	NR_114479.1	AB039451.1	AB039581.1	KX186888.1
*Pseudomonas savastanoi* ATCC 13522^T^	AB021402.1	AB039469.1	AB039514.1	AJ717422.1
*Pseudomonas simiae* CCUG 50988^T^	NR_042392.1	FN554227.1	FN554513.1	FN554757.1
*Pseudomonas stutzeri* LMG 11199^T^	MT027239.1	RHHC01000001.1	KR780035.1	RHHC01000002.1
*Pseudomonas synxantha* LMG 2190^T^	KX186990.1	JN589913.1	JN589943.1	KX186912.1
*Pseudomonas syringae* ATCC 19310^T^	NR_114480.1	NZ_JALK01000001.1	NZ_JALK01000057.1	NZ_JALK01000055.1

**TABLE A2:** Metabolic fingerprints for strain S150 using the Biolog GENIII Microplate™ identification system

Assay	S150	Assay	S150	Assay	S150
Positive Control	+	d‐Arabitol	−	l‐Lactic Acid	+
Dextrin	+	myo‐Inositol	+	Citric Acid	+
d‐Maltose	−	Glycerol	+	α‐Keto‐Glutaric Acid	−
d‐Trehalose	+	d‐Glucose‐6‐PO4	−	d‐Malic Acid	−
d‐Cellobiose	+	d‐Fructose‐6‐PO4	W	Potassium Tellurite	+
Gentiobiose	+	d‐Aspartic Acid	−	Tween 40	−
Sucrose	+	d‐Serine	−	γ‐Amino‐Butryric Acid	−
d‐Turanose	−	Troleandomycin	−	α‐Hydroxy‐Butyric Acid	−
Stachyose	−	Rifamycin SV	−	β‐Hydroxy‐D,L‐butyric Acid	−
pH 6	+	Minocycline	−	α‐Keto‐Butyric Acid	−
pH 5	+	Gelatin	−	Acetoacetic Acid	−
d‐Raffinose	−	Glycyl‐L‐Proline	−	l‐Malic Acid	+
α‐d‐Lactose	−	l‐Alanine	+	Bromo‐Succinic Acid	−
d‐Melibiose	−	l‐Arginine	−	Nalidixic Acid	−
β‐Methyl‐D‐Glucoside	+	l‐Aspartic Acid	+	Lithium Chloride	+
d‐Salicin	−	l‐Glutamic Acid	+	Propionic Acid	−
N‐Acetyl‐D‐Glucosamine	W	l‐Histidine	−	Acetic Acid	−
N‐Acetyl‐β‐D‐Mannosamine	−	l‐Pyroglutamic Acid	−	Formic Acid	−
N‐Acetyl‐D‐Galactosamine	−	l‐Serine	−	Aztreonam	W
N‐Acetyl Neuraminic Acid	−	Lincomycin	−	Sodium Butyrate	+
1% NaCl	+	Guanidine HCl	+	Sodium Bromate	−
4% NaCl	+	Niaproof 4	−	p‐Hydroxy‐Phenylacetic Acid	−
8% NaCl	+	Pectin	+	Methyl Pyruvate	−
α‐d‐Glucose	+	d‐Galacturonic Acid	−	d‐Lactic Acid Methyl Ester	−
d‐Mannose	+	l‐Galactonic Acid	−	Mucic Acid	−
d‐Fructose	+	d‐Gluconic Acid	−	Quinic Acid	−
d‐Galactose	−	d‐Glucuronic Acid	−	d‐Saccharic Acid	−
3‐Methyl Glucose	−	Glucuronamide	−	Vancomycin	−
d‐Fucose	−	Fusidic Acid	−	Tetrazolium Violet	W
l‐Fucose	−	d‐Serine	−	Tetrazolium Blue	−
l‐Rhamnose	−	d‐Sorbitol	+	1% Sodium Lactate	+
Inosine	−	d‐Mannitol	+	Negative Control	−

Abbreviations: (−) = Negative; + = Positive; W = Weak.

**TABLE A3:** The gene clusters of *P. koreensis* S150 specifically contained compared to the other three strains D26, CRS05‐R5, and BS368

Cluster ID	Protein count	Swiss‐Prot hit	GO annotation
cluster503	4	P80011	GO:0006313; P:transposition, DNA‐mediated; IEA:InterPro
cluster4229	3	N/A	N/A
cluster4890	2	Q8R936	GO:0008270; F:zinc ion binding; IEA:UniProtKB‐UniRule
cluster4891	2	N/A	N/A
cluster4892	2	A9VJ02	GO:0000724; P:double‐strand break repair via homologous recombination
cluster4893	2	A2XRZ0	GO:0016491; F:oxidoreductase activity; IEA:UniProtKB‐KW
cluster4894	2	N/A	N/A
cluster4895	2	Q5RBB9	GO:0016491; F:oxidoreductase activity; IEA:UniProtKB‐KW
cluster4896	2	Q3KJK7	GO:0003677; F:DNA binding; IEA:UniProtKB‐KW
cluster4897	2	Q9I310	GO:0071732; P:cellular response to nitric oxide; IMP:PseudoCAP
cluster4898	2	N/A	N/A
cluster4899	2	N/A	N/A
cluster4900	2	N/A	N/A
cluster4901	2	N/A	N/A
cluster4902	2	N/A	N/A
cluster4903	2	N/A	N/A
cluster4904	2	N/A	N/A
cluster4905	2	N/A	N/A
cluster4906	2	N/A	N/A
cluster4907	2	P03710	GO:0019068; P:virion assembly; IEA:InterPro
cluster4908	2	N/A	N/A
